# Loperamide cases reported to United States poison centers, 2010–2022

**DOI:** 10.1186/s40621-023-00473-2

**Published:** 2023-11-24

**Authors:** Aaditya Patel, Natalie I. Rine, Henry A. Spiller, Hannah Hays, Jaahnavi Badeti, Motao Zhu, Kele Ding, Gary A. Smith

**Affiliations:** 1 Center for Injury Research and Policy, The Abigail Wexner Research Institute at Nationwide Children’s Hospital, 700 Children’s Drive, Columbus, OH 43205 USA; 2 Kansas City University College of Osteopathic Medicine, Kansas City, MO USA; 3 Central Ohio Poison Center, Nationwide Children’s Hospital, Columbus, OH USA; 4 Department of Pediatrics, The Ohio State University College of Medicine, Columbus, OH USA; 5 Child Injury Prevention Alliance, Columbus, OH USA

**Keywords:** Loperamide, Poisoning, Abuse, Misuse, Suicide

## Abstract

**Background:**

Intentional use of high doses of loperamide has been linked to serious cardiac toxicity. The objective of this study is to investigate the characteristics and trends of loperamide cases reported to United States (US) poison centers and to evaluate the changes in reported loperamide cases following US Food and Drug Administration (FDA) warnings, labeling requirements, and packaging restrictions for loperamide starting in 2016, with an emphasis on intentional exposures.

**Methods:**

Data from the National Poison Data System were analyzed.

**Results:**

There were 12,987 cases reported to US poison centers from 2010 to 2022, for which, loperamide was the most likely substance responsible for observed clinical effects. Although 46.1% of these cases were associated with minor or no effect, 13.4% resulted in a serious medical outcome, including 59 deaths (0.5%). Eight percent (8.1%) of cases were admitted to a critical care unit and 5.0% were admitted to a non-critical care unit. Among cases with a serious medical outcome, most were associated with loperamide abuse (38.0%), intentional-misuse (15.7%), or suspected suicide (27.5%). The majority (60.0%; n = 33) of fatalities were related to abuse, followed by suspected suicide (20.0%; n = 11) and intentional-misuse (5.5%, n = 3). The rate of loperamide cases per 100,000 US population reported to US PCs decreased from 0.44 in 2010 to 0.36 in 2015 (p = 0.0290), followed by an increase to 0.46 in 2017 (p = 0.0013), and then a trend reversal with a decrease to 0.28 in 2022 (p < 0.0001). The rate of serious medical outcomes related to loperamide increased from 0.03 in 2010 to 0.05 in 2015 (p = 0.0109), which subsequently increased rapidly to 0.11 in 2017 (p < 0.0001), and then demonstrated a trend reversal and decreased to 0.04 in 2022 (p < 0.0001).

**Conclusions:**

FDA warnings, labeling requirements, and packaging restrictions may have contributed to the observed trend reversal and decrease in reports to US poison centers of loperamide cases related to intentional misuse, abuse, and suspected suicide. This demonstrates the potential positive effect that regulatory actions may have on public health. These findings contribute to the evidence supporting the application of similar prevention efforts to reduce poisoning from other medications associated with intentional misuse, abuse, and suicide.

## Background

Loperamide is an anti-diarrheal medication that is available over-the-counter. Structurally similar to haloperidol and diphenoxylate, loperamide functions as an opioid agonist by binding opiate receptors in the intestine, reducing peristalsis and gastric motility (Borron et al. 2017). At a therapeutic dose (8 mg per day maximum for over-the-counter use), loperamide is considered safe with no central opioid effects due to its poor gastrointestinal absorption and limited ability to cross the blood–brain barrier (Borron et al. 2017; US Food and Drug Administration 2016). However, if taken in excessive amounts or concurrently with other medications that increase loperamide concentrations, loperamide can exhibit central opioid effects, including analgesic activity, euphoria, central nervous system depression, and respiratory depression (Borron et al. 2017). Although loperamide was not initially considered a potential drug of abuse when it was approved for over-the-counter sale in the 1976 (US Food and Drug Administration 2016). its misuse and abuse have been reported repeatedly since 2007 (Borron et al. 2017; Vakkalanka et al. 2017; Tringale et al. 2021; Miller et al. 2017; Lasoff et al. 2017; Webb et al. 2020). Individuals may misuse loperamide by taking high doses to treat symptoms of opioid withdrawal or abuse loperamide by taking high doses for a euphoric effect (US Food and Drug Administration 2016; Dierksen et al. 2015).

Importantly, loperamide misuse and abuse have been linked to cardiac toxicity (Wu and Juurlink 2017; Swank et al. 2017), especially with chronic ingestion of high doses (> 100 mg of loperamide per day) (Lasoff et al. 2017). Cardiac effects include QRS widening, prolonged QT interval, Torsades de Pointes, other ventricular dysrhythmias, cardiac arrest, and death (US Food and Drug Administration 2016; Vakkalanka et al. 2017; Wu and Juurlink 2017). In June 2016, the United States (US) Food and Drug Administration (FDA) issued a warning highlighting the risk of these serious cardiac effects when loperamide is taken at doses higher than recommended (US Food and Drug Administration 2016). This initial warning was followed by numerous updates through September 2019 by the FDA that announced labeling requirements and packaging restrictions for loperamide (US Food and Drug Administration 2016, 2018).

The most recent study to evaluate the epidemiology of loperamide cases reported to US poison centers (PCs) included data only through 2015 (Vakkalanka et al. 2017), which predates the FDA loperamide actions. Therefore, additional research is needed to evaluate the influence of the FDA’s interventions on loperamide exposure trends. The objective of this study is to update our understanding of the characteristics and trends of loperamide cases reported to US PCs and to evaluate the changes in reported loperamide cases following the FDA warnings, labeling requirements, and packaging restrictions for loperamide starting in 2016, with an emphasis on intentional misuse, abuse, and suicidal behavior.

## Methods

### Data source

The National Poison Data System (NPDS) collects data from calls to US PCs in near real-time. The NPDS is operated by America’s Poison Centers, formerly known as the American Associations of Poison Control Centers (Gummin et al. 2021). This study retrospectively analyzed data regarding loperamide exposure cases reported to the NPDS from January 1, 2010 through December 31, 2022. Exposure cases are reports involving contact with the substance and not reports of calls seeking information about the substance without contact. The terms “case” and “exposure” are used interchangeably in this article when referring to these exposure cases reported to PCs. US population estimates were obtained from the US Census Bureau to calculate age-group-specific and sex-specific population-based rates (United States Census Bureau 2023). This study was determined to be exempt from approval by the institutional review board at the authors’ institution.

### Case selection

Data were obtained from the NPDS for reported cases involving loperamide using its generic code (0550000). Cases with a medical outcome categorized by the NPDS as “confirmed non-exposure” (n = 89) or “unrelated effect” (n = 743) or a reason for exposure categorized by the NPDS as “unintentional-food poisoning” (n = 13) or “adverse reaction – food” (n = 5) were excluded from the study. The NPDS defines “confirmed non-exposure” as a case for which “there is reliable and objective evidence that the exposure never occurred and that any symptoms exhibited by the patient were not related to the reported exposure,” and “unrelated exposure” as an exposure for which “based upon all the information available, the exposure was probably not responsible for the effect.”(National Poison Data System Coding Users’ 2014) America’s Poison Centers has a three-step review process for fatal cases. One objective of this review process is to assign a relative contribution to fatality value for involved substances: (1) undoubtedly responsible, (2) probably responsible, (3) contributory, (4) probably not responsible, (5) undoubtedly not responsible, and (6) unknown. Seven deaths were excluded from this study which had a relative contribution to fatality indicating that loperamide was “probably not responsible” or “undoubtedly not responsible” (National Poison Data System 2023).

### Study variables

Subjects in the study were grouped by sex (male and female) and age (< 6 years, 6–19 years, 20–29 years, 30–39 years, and > 39 years). Exposure type was categorized into single-substance and multiple-substance exposures based on the number of substances involved. For the analyses, reason for exposure was categorized as (1) unintentional, which includes unintentional-general, unintentional-therapeutic error, unintentional-misuse, unintentional-other (including unintentional-environmental, unintentional-occupational, and unintentional-unknown); (2) intentional, which includes intentional-suspected suicide, intentional-misuse, intentional-abuse, and intentional-unknown; (3) other, and (4) unknown reason for exposure. The NPDS defines unintentional-misuse as “unintentional improper or incorrect use of a non-pharmaceutical substance. Unintentional-misuse differs from intentional-misuse in that the exposure was unplanned or not foreseen by the patient.” The NPDS defines intentional-misuse as “an exposure resulting from the intentional improper or incorrect use of a substance for reasons other than the pursuit of a psychotropic effect,” and intentional-abuse as “an exposure resulting from the intentional improper or incorrect use of a substance where the patient was likely attempting to gain a high, euphoric effect or some other psychotropic effect, including recreational use of a substance for any effect” (National Poison Data System Coding Users 2014).

NPDS categorizes highest level of health care received into: (1) no health care facility (HCF) treatment received, (2) treated/evaluated and released, (3) admitted to a critical care unit (CCU), 4) admitted to a non-CCU, (5) admitted to a psychiatric facility, (6) patient refused or did not arrive at a HCF, and (7) patient lost to follow-up/left against medical advice/unknown. The “no HCF treatment received” category was derived as the sum of the “managed on-site (not in a HCF)” and “other” categories of the NPDS management site variable. If the management site was “unknown,” then that case was included in the “lost to follow-up/ left against medical advice/ unknown” category of the highest level of health care received variable. For analyses, “lost to follow-up/ left against medical advice/ unknown” was considered as unknown. In addition, during some of the analyses, admitted to a CCU or non-CCU were combined into a single category representing medical-related admissions.

Medical outcomes are grouped by the NPDS into: (1) no effect (no symptoms developed as a result of the exposure), (2) minor effect (symptoms that usually resolve rapidly without residual disability or disfigurement), (3) moderate effect (symptoms which are more pronounced, prolonged, or systemic than minor symptoms), (4) major effect (symptoms are life-threatening or result in significant disability or disfigurement), (5) death, (6) not followed (includes minimal clinical effects possible and judged as a non-toxic exposure), and (7) unknown (includes unable to follow [judged as potentially toxic exposure]). For analyses, moderate effect, major effect, and death were combined into a “serious medical outcome” category.

### Statistical analysis

Data were analyzed using IBM SPSS 28.0 (IBM Corporation, Armonk, NY) and SAS 9.4 (SAS Institute, Inc. Cary, NC). US Census data for 2010–2022 (including age group-specific and sex-specific estimates) were used to calculate national loperamide exposure rates, which are expressed per 100,000 population in this article.^13^ The statistical significance of trends over time was evaluated by using simple or piecewise linear regression analysis, as appropriate. The level of statistical significance was α = 0.05. In addition, risk ratios (RRs) were calculated with 95% confidence intervals (CIs).

Analyses of the general characteristics of loperamide cases included only those in which loperamide was the first-ranked substance, which is defined by the NPDS as the substance judged by a Specialist in Poison Information at the PC to be most likely responsible for the observed clinical effects. Cases in which loperamide is first-ranked include (1) all single-substance (loperamide only) exposures and (2) multiple-substance exposures in which loperamide is the first-ranked substance. All loperamide cases reported to US PCs (including first-ranked and non-first-ranked) were included in analyses of trends over time. Non-first-ranked exposures were included in analyses of trends because we wanted to capture the magnitude of loperamide’s presence in these trends over time; however, the associations of loperamide with characteristics, such as medical outcome and admission to a CCU or non-CCU, were evaluated with first-ranked cases because attribution of the observed associations to loperamide would be clearer when loperamide was judged to be the most likely substance responsible for the observed clinical effects.

## Results

### General characteristics

There were 12,987 cases involving loperamide as the first-ranked substance reported to US PCs from 2010 through 2022, averaging 999 cases annually. Children < 6 years old accounted for nearly half of the cases (48.1%; with a peak at age 2 years), followed by individuals > 39 years old (25.2%) (Table 1). Although 52.5% of cases occurred among females overall, there was a male predominance among 20–29-year-olds (63.1%) and 30–39-year-olds (57.9%) (Table 1). Most (86.6%) cases involved only the single substance of loperamide, and most occurred in a residence (97.6%) (data not shown in table).

**Table 1:** Characteristics of First-Ranked Loperamide Cases Reported to US Poison Centers by Age Group, National Poison Data System 2010–2022

	Age groups						
Characteristics	< 6 Years n (%)^a^	6–19 Years n (%)^a^	20–29 Years n (%)^a^	30–39 Years n (%)^a^	> 39 Years n (%)^a^	Unknown n	Total n (%)^a^
*Sex*							
Male	3,018 (50.9)	455 (43.3)	758 (63.1)	609 (57.9)	1,088 (35.0)	222	6,150 (47.5)
Female	2,910 (49.1)	595 (56.7)	444 (36.9)	442 (42.1)	2,020 (65.0)	376	6,787 (52.5)
Unknown	11	1	1	3	4	30	50
*Exposure type*							
Single-substance	5,447 (91.7)	781 (74.3)	917 (76.2)	824 (78.2)	2,706 (86.9)	573	11,248 (86.6)
Multiple-substance	492 (8.3)	270 (25.7)	286 (23.8)	230 (21.8)	406 (13.1)	55	1,739 (13.4)
*Highest level of health care received*							
No HCF treatment received	4,048 (71.2)	485 (49.4)	293 (27.1)	286 (29.5)	1,703 (60.2)	453	7,268 (60.3)
Treated/ evaluated and released	1,307 (23.0)	199 (20.3)	244 (22.6)	224 (23.1)	447 (15.8)	14	2,435 (20.2)
Admitted to a HCF	171 (3.0)	264 (26.9)	495 (45.7)	420 (43.3)	576 (20.4)	8	1,934 (16.0)
CCU	50 (0.9)	60 (6.1)	299 (27.6)	280 (28.8)	288 (10.2)	2	979 (8.1)
Non-CCU	121 (2.1)	62 (6.3)	110 (10.2)	82 (8.4)	230 (8.1)	2	607 (5.0)
Psychiatric facility	0 (0.0)	142 (14.5)	86 (8.0)	58 (6.0)	58 (2.1)	4	348 (2.9)
Patient refused referral/did not arrive at a HCF	156 (2.8)	33 (3.4)	50 (4.6)	41 (4.2)	104 (3.7)	39	423 (3.5)
Patient lost to follow-up/Left against medical advice/Unknown	257	70	121	83	282	114	927
*Medical outcome*							
No effect	2,957 (52.9)	317 (32.5)	145 (13.4)	127 (13.2)	517 (18.0)	53	4,116 (34.4)
Minor effect	329 (5.9)	199 (20.4)	224 (20.7)	151 (15.7)	460 (16.0)	37	1,400 (11.7)
Serious medical outcome^b^	39 (0.7)	115 (11.8)	463 (42.7)	436 (45.3)	536 (18.6)	14	1,603 (13.4)
Moderate effect	34 (0.6)	95 (9.7)	281 (25.9)	265 (27.6)	375 (13.0)	14	1,064 (8.9)
Major effect	5 (0.1)	17 (1.7)	154 (14.2)	152 (15.8)	152 (5.3)	0	480 (4.0)
Death	0 (0.0)	3 (0.3)	28 (2.6)	19 (2.0)	9 (0.3)	0	59 (0.5)
Not followed^c^	2,263 (40.5)	344 (35.3)	252 (23.3)	248 (25.8)	1,368 (47.5)	382	4,857 (40.6)
Unable to follow^d^	351	76	119	92	231	142	1,011
*Reason for exposure*							
Unintentional	5,900 (99.4)	478 (46.1)	240 (20.3)	246 (23.7)	1,652 (54.1)	339	8,855 (68.9)
Unintentional-General	5,577 (94.0)	193 (18.6)	56 (4.7)	62 (6.0)	351 (11.5)	121	6,360 (49.5)
Unintentional-Therapeutic error	309 (5.2)	240 (23.2)	152 (12.9)	147 (14.2)	1,134 (37.1)	187	2,169 (16.9)
Unintentional – Misuse	12 (0.2)	40 (3.9)	29 (2.5)	34 (3.3)	156 (5.1)	30	301 (2.3)
Unintentional – Other^e^	2 (0.0)	5 (0.5)	3 (0.3)	3 (0.3)	11 (0.4)	1	25 (0.2)
Intentional	6 (0.1)	497 (48.0)	850 (72.0)	724 (69.9)	1,131 (37.0)	152	3,360 (26.1)
Suspected suicide	0 (0.0)	356 (34.4)	261 (22.1)	175 (16.9)	288 (9.4)	26	1,106 (8.6)
Intentional – Misuse	6 (0.1)	53 (5.1)	178 (15.1)	186 (18.0)	592 (19.4)	77	1,092 (8.5)
Abuse	0 (0.0)	62 (6.0)	368 (31.2)	325 (31.4)	183 (6.0)	34	972 (7.6)
Intentional—Unknown	0 (0.0)	26 (2.5)	43 (3.6)	38 (3.7)	68 (2.2)	15	190 (1.5)
Other	27 (0.5)	61 (5.9)	91 (7.7)	66	271 (6.4)	124	640 (5.0)
Unknown	6	15	22	18	58	13	132
Total (row %)	5,939 (48.1)	1,051 (8.5)	1,203 (9.7)	1,054 (8.5)	3,112 (25.2)	628	12,987 (100.0)

CCU—Critical Care Unit, HCF—Health Care Facility

^a^Column percentages may not add to 100.0% due to rounding error

^b^Includes moderate effect, major effect, and death

^c^Not followed (minimal clinical effects possible)

^d^Unable to follow (judged as a potentially toxic exposure)

^e^Includes unintentional-environmental, unintentional-occupational, and unintentional-unknown

### Medical outcome and highest level of health care received

Although more than one-third (34.4%) of reported cases involving loperamide as the first-ranked substance were associated with no effect and 11.7% were associated with a minor effect, 13.4% of cases experienced a serious medical outcome, including 59 deaths (0.5%). Most serious medical outcomes occurred among 20–29-year-olds (42.7%) and 30–39-year-olds (45.3%), while only 0.7% (n = 39) were among children < 6 years old. Almost half (47.5%, n = 28) of the deaths were experienced by 20–29-year-olds.

Most reported cases involving loperamide as the first-ranked substance did not receive treatment in a HCF (60.3%) or were treated/evaluated and released (20.2%); however, 8.1% were admitted to a CCU and 5.0% were admitted to a non-CCU. Individuals 20–29 years old accounted for 27.6% of admissions to a CCU and individuals 30–39 years old accounted for 28.8%, while children < 6 years old were involved in only 0.9% of CCU admissions (Table 1). Individuals > 19 years old were 12 times more likely to experience a serious medical outcome (RR: 12.4, 95% CI 10.6–14.6) and 6 times more likely to be admitted to either a CCU or non-CCU (RR: 6.0, 95% CI 5.3–6.8) than younger individuals. Multiple-substance exposures were 3 times more likely to be associated with a serious medical outcome (RR: 3.0, 95% CI 2.7–3.3) or admission to either a CCU or non-CCU (RR: 2.9, 95% CI 2.6–3.1) than single-substance exposures.

### Reason for exposure

The reason for exposure for approximately half (49.5%) of the reported cases involving loperamide as the first-ranked substance was “unintentional-general,” followed by “unintentional-therapeutic errors” (16.9%) (Table 1). Intentional reasons were also common and included suspected suicide (8.6%), intentional-misuse (8.5%), and abuse (7.6%). Nearly all (99.4%) reported exposures among < 6-year-olds and more than half (54.1%) among > 39-year-olds were unintentional. Among children < 6 years old, 94.0% of exposures were associated with “unintentional-general,” which represents exploratory behaviors in this age group. Among individuals > 39 years old, therapeutic errors predominated (37.1%). Among the other age groups, intentional reasons for exposure were common (6–19 years: 48.0%; 20–29 years: 72.0%; 30–39 years: 69.9%), with suspected suicide accounting for more than one-third (34.4%) of reported cases among 6–19-year-olds, and loperamide abuse accounting for almost one-third of cases among 20–29-year-olds (31.2%) and 30–39-year-olds (31.4%) (Table 1).

Among reported cases with a serious medical outcome, most were associated with loperamide abuse (38.0%), intentional-misuse (15.7%), or suspected suicide (27.5%) (Table 2). Among the fatalities, the majority were related to abuse (60.0%; n = 33), followed by suspected suicide (20.0%; n = 11) and intentional-misuse (5.5%, n = 3). In addition, among reported cases associated with admission to a CCU, most were related to abuse (41.6%), intentional-misuse (13.0%), or suspected suicide (28.4%). Among single-substance exposures, more than half (52.0%) were associated with unintentional-general exposures, followed by therapeutic errors (18.3%), and intentional-misuse (8.7%). Similarly, among multiple-substance exposures, almost one-third (32.9%) were associated with unintentional-general exposures, followed by suspected suicide (30.6%) and abuse (12.2%) (Table 2). Abuse-related cases were more likely to result in a serious medical outcome (RR: 7.9, 95% CI: 7.4–8.5) or admission to either a CCU or non-CCU (RR: 6.5, 95% CI: 6.0–7.1) than non-abuse-related cases.

**Table 2:** Characteristics of First-Ranked Loperamide Cases Reported to US Poison Centers by Reason for Exposure, National Poison Data System 2010–2022

Characteristics	Unintentional reasons				Intentional reasons						
	General n (%)^a^	Therapeutic Error n (%)^a^	Misuse n (%)^a^	Other^f^ n (%)^a^	Suspected Suicide n (%)^a^	Misuse n (%)^a^	Abuse n (%)^a^	Unknown n (%)^a^	Other n (%)^a^	Unknown n	Total n (%)^b^
*Sex*											
Male	3,111 (51.1)	891 (14.6)	139 (2.3)	6 (0.1)	436 (7.2)	497 (8.2)	676 (11.1)	91 (1.5)	246 (4.0)	57	6,150 (47.5)
Female	3,224 (48.0)	1,273 (19.0)	162 (2.4)	18 (0.3)	664 (9.9)	591 (8.8)	293 (4.4)	96 (1.4)	394 (5.9)	72	6,787 (52.5)
Unknown	25	5	0	1	6	4	3	3		3	50
*Exposure type*											
Single-substance	5,796 (52.0)	2,043 (18.3)	279 (2.5)	21 (0.2)	581 (5.2)	965 (8.7)	762 (6.8)	149 (1.3)	544 (4.9)	108	11,248 (86.6)
Multiple-substance	564 (32.9)	126 (7.3)	22 (1.3)	4 (0.2)	525 (30.6)	127 (7.4)	210 (12.2)	41 (2.4)	96 (5.6)	24	1,739 (13.4)
*Highest level of health care received*											
No HCF treatment received	4,342 (59.9)	1,845 (25.5)	195 (2.7)	8 (0.1)	6 (0.1)	351 (4.8)	46 (0.6)	29 (0.4)	424 (5.9)	22	7,268 (60.3)
Treated/ evaluated and released	1,344 (55.5)	159 (6.6)	45 (1.9)	4 (0.2)	276 (11.4)	278 (11.5)	221 (9.1)	30 (1.2)	63 (2.6)	15	2,435 (20.2)
Admitted to a HCF	205 (10.9)	44 (2.3)	21 (1.1)	11 (0.6)	687 (36.7)	236 (12.6)	551 (29.4)	87 (4.6)	32 (1.7)	60	1,934 (16.0)
CCU	61 (6.5)	15 (1.6)	7 (0.7)	7 (0.7)	268 (28.4)	123 (13.0)	393 (41.6)	55 (5.8)	15 (1.6)	35	979 (8.1)
Non-CCU	133 (22.7)	28 (4.8)	12 (2.1)	4 (0.7)	161 (27.5)	93 (15.9)	118 (20.2)	23 (3.9)	13 (2.2)	22	607 (5.0)
Psychiatric facility	11 (3.2)	1 (0.3)	2 (0.6)	0 (0.0)	258 (74.8)	20 (5.8)	40 (11.6)	9 (2.6)	4 (1.2)	3	348 (2.9)
Patient refused referral/did not arrive at a HCF	173 (41.8)	41 (9.9)	13 (3.1)	2 (0.5)	41 (9.9)	63 (15.2)	38 (9.2)	15 (3.6)	28 (6.8)	9	423 (3.5)
Patient lost to follow-up/Left against medical advice/Unknown	296	80	27	0	96	164	116	29	93	26	927
*Medical outcome*											
No effect	3,016 (73.6)	434 (10.6)	57 (1.4)	2 (0.0)	286 (7.0)	188 (4.6)	69 (1.7)	20 (0.5)	28 (0.7)	16	4,116 (34.4)
Minor effect	366 (26.5)	204 (14.8)	41 (3.0)	3 (0.2)	245 (17.7)	207 (15.0)	139 (10.1)	30 (2.2)	146 (10.6)	19	1,400 (11.7)
Serious medical outcome^c^	73 (4.7)	40 (2.6)	27 (1.7)	11 (0.7)	426 (27.5)	243 (15.7)	589 (38.0)	78 (5.0)	64 (4.1)	52	1,603 (13.4)
Moderate effect	65 (6.3)	34 (3.3)	19 (1.8)	4 (0.4)	288 (27.7)	191 (18.4)	342 (32.9)	38 (3.7)	57 (5.5)	26	1,064 (8.9)
Major effect	8 (1.7)	6 (1.3)	6 (1.3)	7 (1.5)	127 (27.7)	49 (10.7)	214 (46.7)	34 (7.4)	7 (1.5)	22	480 (4.0)
Death	0 (0.0)	0 (0.0)	2 (3.6)	0 (0.0)	11 (20.0)	3 (5.5)	33 (60.0)	6 (10.9)	0 (0.0)	4	59 (0.5)
Not followed^d^	2,508 (51.8)	1,418 (29.3)	162 (3.3)	6 (0.1)	27 (0.6)	301 (6.2)	54 (1.1)	26 (0.5)	337 (7.0)	18	4,857 (40.6)
Unable to follow^e^	397	73	14	3	122	153	121	36	65	27	1,011
*Total (row %)*	6,360 (49.5)	2,169 (16.9)	301 (2.3)	25 (0.2)	1,106 (8.6)	1,092 (8.5)	972 (7.6)	190 (1.5)	640 (5.0)	132	12,987 (100.0)

CCU—Critical Care Unit, HCF—Health Care Facility

^a^Row percentage may not add to 100.0% due to rounding error

^b^Column percentage may not add up to 100.0% due to rounding error

^c^Includes moderate effect, major effect, and death

^d^Not followed (minimal clinical effects possible)

^e^Unable to follow (judged as a potentially toxic exposure)

^f^Includes unintentional-environmental, unintentional-occupational, and unintentional-unknown

### Fatalities

Among the 59 reported deaths associated with loperamide as the first-ranked substance, the relative contribution to fatality for loperamide was determined to be undoubtedly responsible for 17 deaths, probably responsible for 20 deaths, contributory for 3 deaths, and unknown for 3 deaths; the relative contribution to fatality was missing for 16 deaths. Most fatalities occurred among males (71.2%, n = 42), and the 20–29-year-old and 30–39-year-old age groups combined accounted for 79.7% (n = 47) of deaths. More than half (55.9%, n = 33) of the deaths were associated with abuse, followed by suspected suicide (18.6%, n = 11). Single-substance exposures accounted for 52.5% (n = 31) of fatalities (data not shown in table). Among these single-substance exposures, the following selected cardiac effects related to loperamide were documented: ventricular tachycardia/ventricular fibrillation (n = 9), QTC prolongation (n = 8), QRS prolongation (n = 7), and Torsades de Pointes (n = 2). Notably, multiple-substance exposures were almost six times more likely to be associated with death (RR: 5.7, 95% CI: 3.4–9.5) than single-substance exposures. Among multiple-substance exposures, the substances most commonly documented in addition to loperamide were benzodiazepines (n = 7), ethanol beverages (n = 5), and diphenhydramine (n = 3). Individuals > 19 years old were approximately 25 times more likely to experience death (RR: 24.9, 95% CI: 7.8–79.4) than younger individuals. Cases related to abuse were 19 times more likely to be associated with fatality (RR: 19.4, 95% CI: 11.4–33.2) than non-abuse cases.

### Trends

**Fig. 1:**
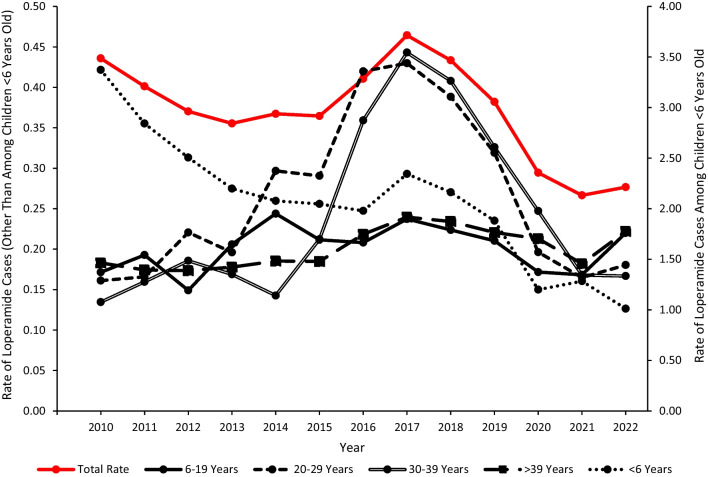
Annual rate per 100,000 US population of loperamide cases reported to US poison centers by age group, National Poison Data System 2010–2022

Trend analyses included all 15,509 loperamide cases (both first-ranked and non-first-ranked) reported to US PCs from 2010 through 2022. There was an average of 1,193 cases annually; however, the trend in annual frequency varied during the study period. There were 1,348 loperamide-related cases in 2010, which decreased by 13.3% to 1,169 exposures in 2015, followed by an increase of 29.1% to 1,509 in 2017, and then a decrease of 38.9% to 922 cases in 2022. The rate of loperamide exposures per 100,000 US population reported to US PCs followed a similar trend pattern, which demonstrated a decrease from 0.44 in 2010 to 0.36 in 2015 (p = 0.0290), followed by an increase to 0.46 in 2017 (p = 0.0013) and then a decrease to 0.28 in 2022 (p < 0.0001) (Fig. 1). Children < 6 years old consistently experienced higher loperamide exposure rates than the other age groups and were primarily responsible for the trend pattern for all individuals during the first half of the study period. During the latter half of the study period, the trend pattern for all individuals was primarily influenced by a combination of < 6-year-olds, 20–29-year-olds, and 30–39-year-olds. The rate of loperamide exposure among children < 6 years old decreased from 3.37 in 2010 to 1.98 in 2016 (p = 0.0002), followed by an increase to 2.34 in 2017 (p = 0.0135), and then decreased to 1.01 in 2022 (p = 0.0002). The rate of loperamide exposure among 30–39-year-olds did not change significantly from 0.13 in 2010 to 0.14 in 2014 (p = 0.8660), but then increased to 0.44 in 2017 (p < 0.0001), followed by a decline to 0.17 in 2022 (p < 0.0001). The rate of loperamide exposure among 20–29-year-olds showed an increase from 0.16 in 2010 to 0.43 in 2017 (p < 0.0001), followed by a decrease to 0.18 in 2022 (p < 0.0001) (Fig. 1). The overall trend patterns were similar for males and females, with females experiencing higher exposure rates than males, except in 2017.

**Fig. 2:**
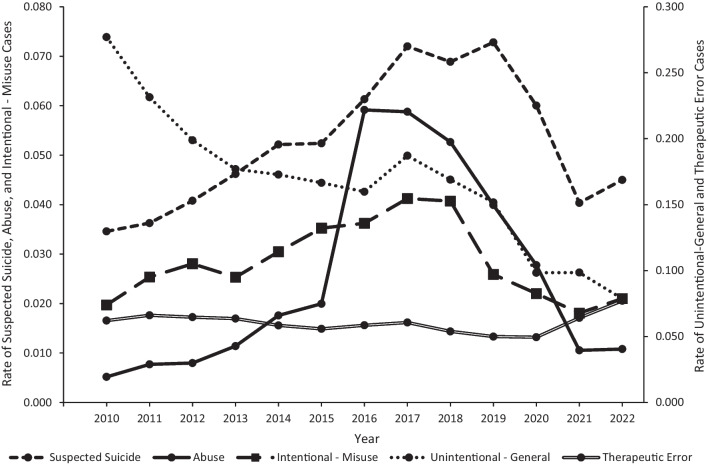
Annual rate per 100,000 US population of Loperamide cases reported to US poison centers by reason for exposure, National Poison Data System 2010–2022

Consistent with the age group trends, the reason for exposure that contributed the most to the trend pattern for reported loperamide exposures during the first half of the study period was unintentional-general; the trend pattern during the latter half of the study period was influenced by a combination of unintentional-general, suspected suicide, and abuse (Fig. 2). The rate of loperamide exposure associated with an unintentional-general reason for exposure decreased from 0.28 in 2010 to 0.16 in 2016 (p = 0.0002), followed by an increase to 0.19 in 2017 (p = 0.0204), and then decreased to 0.08 in 2022 (p = 0.0002). The rate of suspected suicide cases increased from 0.04 in 2010 to 0.07 in 2017 (p < 0.0001), plateaued until 2019 (p = 0.5353), then decreased to 0.05 in 2022 (p = 0.0005). Likewise, the rate of loperamide exposure associated with abuse increased from 0.005 in 2010 to 0.02 in 2015 (p = 0.0162), then exhibited a sharp rise to 0.06 in 2016 (p < 0.0001), followed by a decrease to 0.01 in 2022 (p < 0.0001). The rate of therapeutic errors decreased from 0.06 in 2010 to 0.05 in 2020 (p = 0.0002), followed by a rapid increase to 0.08 in 2022 (p < 0.0001) (Fig. 2).

**Fig. 3:**
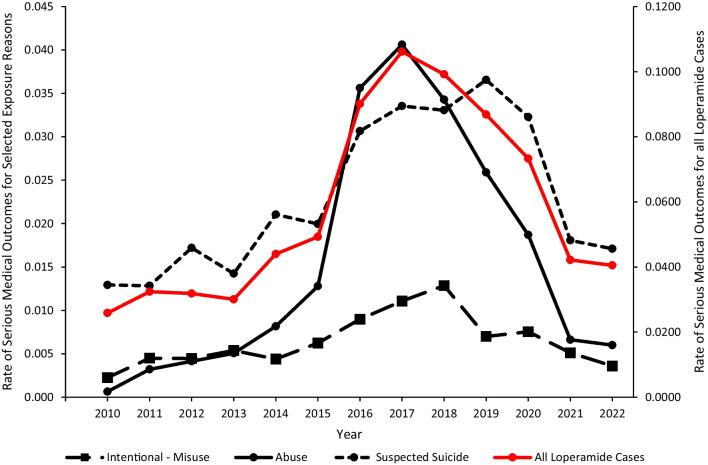
Annual rate per 100,000 US population of serious medical outcomes associated with loperamide cases reported to US poison centers with separate rates for intentional-misuse, abuse, and suspected suicide, National Poison Data System 2010–2022

The rate of reported loperamide exposures associated with a serious medical outcome increased from 0.03 in 2010 to 0.05 in 2015 (p = 0.0109), which subsequently demonstrated a rapid increase to 0.11 in 2017 (p < 0.0001), followed by a decrease to 0.04 in 2022 (p < 0.0001). The rate of a serious medical outcome associated with reported loperamide abuse mirrored this trend pattern (Fig. 3). The trend pattern for serious medical outcome was influenced most by the trends for 20–29-year-olds and 30–39-year-olds.

**Fig. 4:**
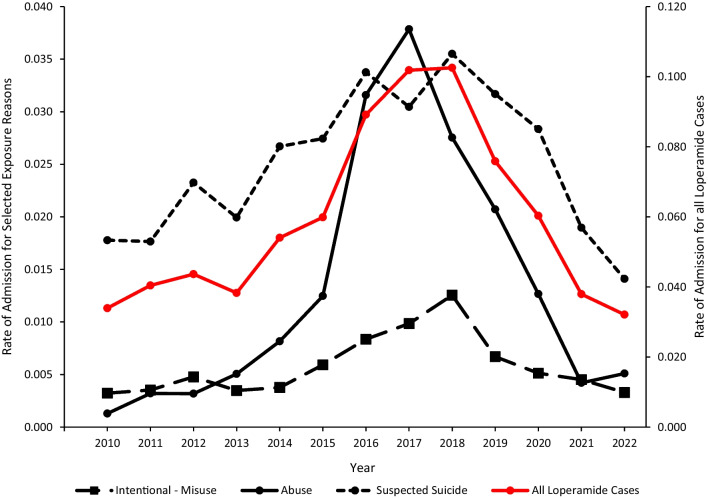
Annual Rate per 100,000 US population of admission to a critical care unit or non-critical care unit (combined) associated with loperamide cases reported to US poison centers with separate rates for intentional-misuse, abuse, and suspected suicide, National Poison Data System 2010–2022

The rate of admission to a CCU or non-CCU (combined) associated with reported loperamide exposure increased from 0.03 in 2010 to 0.10 in 2018 (p < 0.0001), followed by a decrease to 0.03 in 2022 (p < 0.0001) (Fig. 4). This trend was influenced primarily by the rates of CCU or non-CCU admissions (combined) for 1) suspected suicide, which increased from 0.02 in 2010 to 0.04 in 2018 (p < 0.0001), followed by a decrease to 0.01 in 2022 (p < 0.0001) and 2) abuse, which increased from 0.001 in 2010 to 0.04 in 2017 (p < 0.0001), followed by a decrease to 0.005 in 2022 (p = 0.0005). The rate of CCU or non-CCU admissions (combined) associated with suspected suicide was higher than that associated with abuse throughout the study period, except in 2017 (Fig. 4). When admission to the highest level of care (admission to a CCU) was evaluated separately, the trend pattern over time was similar to that for admission to a CCU or non-CCU (combined), except that admission to a CCU peaked one year earlier (2017) (data not shown in figures).

## Discussion

A number of studies have documented an increase in intentional loperamide misuse and abuse during the past decade, including Vakkalanka and colleagues, who reported a 91% increase from 2010 to 2015 (Vakkalanka et al. 2017). Miller et al. reported a surge in published case reports of loperamide misuse and abuse between 2014 and 2016; among the 54 case reports from 1985 to 2016, 33 were between 2014 and 2016 (Miller et al. 2017). Lasoff and colleagues analyzed the California Poison Control Systems database from 2002 to 2015 and identified an increase in intentional loperamide exposures that presented to or were referred to a HCF in 2014 and 2015 along with an increase in the number of cases with cardiotoxicity at supratherapeutic dosages (Lasoff et al. 2017). Borron, et al. conducted a retrospective review of Texas Poison Control Center and NPDS data from 2009 to 2015, showing increasing cardiotoxicity rates linked to intentional loperamide misuse and abuse (Borron et al. 2017).

In June 2016, the US FDA issued a warning highlighting the risk of serious cardiac effects when loperamide is taken at higher-than-recommended doses (US Food and Drug Administration 2016). In updates in November 2016 and March 2017, it announced product labeling changes to help address this problem (US Food and Drug Administration 2016). In January 2018, the FDA announced that it was working with manufacturers on loperamide packaging changes, which culminated in an update in September 2019 announcing its approval of packaging changes for loperamide tablets and capsules, which limited cartons of loperamide to 48 mg and required unit-dose packaging of tablets and capsules (US Food and Drug Administration 2018).

Previous studies that reported an increase in loperamide intentional misuse, abuse, and associated cardiotoxicity did not include data beyond 2015, which predates the FDA’s loperamide warnings, labeling, and packaging restrictions (US Food and Drug Administration 2016, 2018). Our study not only corroborated the previously reported increasing trend, it revealed a reversal of the upward trend in reported intentional loperamide-related exposures starting in 2018, which corresponds temporally with the FDA actions. These findings are consistent with those of another study, which used online social listening technology from 2015 to 2021 and showed a peak in online posts related to loperamide misuse and abuse in 2016 with a subsequent decline in 2019 and 2020 (Tringale et al. 2021).

The loperamide exposure trends observed in this study were likely influenced by numerous factors in addition to the loperamide-specific actions by the FDA. Following the FDA warnings, loperamide manufacturers initiated an educational campaign to inform health professionals about potential loperamide toxicity associated with misuse and abuse (Consumer Healthcare Products Association 2023). The national opioid crisis and associated prevention responses also may have influenced loperamide trends. The increase in opioid use in the US, especially use of illicit fentanyl, which coincided with the beginning of our study period may have contributed to the observed increase in intentional loperamide misuse and abuse. In October 2017, the federal government declared the opioid crisis a public health emergency. This was associated with the development of new abuse-deterrent opioid formulations by the FDA and pain treatment standards by the Joint Commission. In 2016, the FDA voted to require opioid-focused continuing education for all physicians (Jones et al. 2018). Although availability remains inadequate (Jones et al. 2023), the expansion of opioid treatment programs and telehealth access may have helped prevent individuals with opioid use disorder from engaging in loperamide misuse and abuse during the later study years. While it is possible that these initiatives to combat the national opioid crisis may have influenced loperamide exposures, the opioid crisis has continued to grow in contrast to the dramatic reversal in the rates of reported intentional loperamide exposures in this study.

### Unintentional-general exposures among young children

Among the 12,987 cases involving loperamide as the first-ranked substance reported to US PCs from 2010 through 2022, almost half occurred among children < 6 years old, peaking at age 2 years. A vast majority of these cases were single-substance exposures and associated with child exploratory behaviors (unintentional-general exposures), which are typical characteristics of poisoning among young children as they gain increased mobility and probe their environment while putting objects in their mouth without an understanding of danger. Fortunately, most reported loperamide exposures in this age group were associated with a medical outcome of no or minor effect. Prevention measures for this age group must address their unique developmental characteristics. Child-resistant packaging and education of parents and caregivers about safe storage practices, especially in the home, are key prevention strategies. Because the FDA packaging requirements of 2018 limited the number of doses in cartons of loperamide to a total of 48 mg and required unit-dose packaging (such as blister packs) of tablets and capsules, it would be expected that this could decrease access of these products to young children and thereby decrease exposures. This was observed when iron poisoning decreased among young children following the introduction of unit-dose packaging of iron supplements (Tenenbein 2005). Our findings reveal an overall downward trend in reported unintentional-general exposures during the study period with some plateauing from 2013 to 2016, an increase from 2016 to 2017, and then a decline to 2022 (Fig. 2). The decline after 2017 may have been influenced by the FDA actions; however, the overall declining trend in this age group since 2010, as well as the design of the study, preclude causal determinations.

### Suspected suicide

Suspected suicide involving loperamide as the first-ranked substance was an important reason for exposure in this study, accounting for 34% of reported cases among 6–19-year-olds, 22% of cases among 20–29-year-olds, and 17% of cases among 30–39-year-olds. There was a reversal of the upward trend in reported loperamide-related suspected suicide in 2017, which was temporally associated with the implementation of the FDA actions. It is plausible (although not conclusive) that these changes were causally related, based on similar observed reductions in suicide associated with packaging restrictions for over-the-counter acetaminophen implemented in the United Kingdom (Greene et al. 2006; Hawton et al. 2013, 2001, 2011). Hawton and colleagues showed that legislation in England and Wales in 1998 requiring reduced acetaminophen package sizes (acetaminophen availability was mostly limited to blister packs already) was followed by a decrease in acetaminophen-related deaths associated with suicide or undetermined intent (Hawton et al. 2013, 2001). These findings agree with those of Turvill, et al., who also showed that benzodiazepine overdoses, in contrast, remained stable following enactment of the legislation (Turvill et al. 2000). Limited package size may reduce the severity of loperamide-related suicide events by limiting loperamide availability in the home. In addition, because suicidal ingestion is often a highly impulsive act, unit-dose packaging may act as a deterrent by slowing the extraction of the medication (Turvill et al. 2000; Hawton and James 2005).

### Serious medical outcome and admission to a critical care unit or non-critical care unit

Although most loperamide exposures had minimal consequences, an important minority (13.4%) of reported cases involving loperamide as the first-ranked substance were associated with a serious medical outcome (including 59 deaths), with most of these cases occurring among young adults 20–39 years old. Intentional reasons for exposure accounted for more than two-thirds of reported cases in the 20–29-year-old and 30–39-year-old age groups, with loperamide abuse predominating. The rates of loperamide-related serious medical outcomes overall and those associated with abuse increased until 2017 and then declined starting in 2018 (Fig. 3). A similar temporal pattern was observed for the rate of loperamide-related admission to a CCU or non-CCU (combined), with most of these reported cases associated with abuse or suspected suicide. The rate of loperamide-related admission to a CCU or non-CCU (combined) for all reasons for exposure, and the specific admission rates for abuse, suspected suicide, and intentional-misuse all increased until 2017 or 2018, followed by an abrupt trend reversal and decrease (Fig. 4). These temporal trends coincide with the loperamide warnings, labeling requirements, and packaging restrictions by the FDA starting in 2016.

Despite the decline in adverse outcomes following the FDA interventions, the preventable nature of loperamide-related cardiotoxicity and death calls into question whether these actions are enough. A recent survey of pharmacies in all US states demonstrated that while the majority of pharmacists are aware that loperamide is misused, only 30.7% feel that they could restrict sale if misuse is suspected, and only 3.2% had already placed loperamide behind the counter or had a quantity restriction (Feldman and Everton 2020). While ensuring that patients with legitimate medical needs have access to the drug, federal legislation should be considered to further minimize misuse and abuse to prevent cardiotoxicity and death. Practically, this could look similar to The Combat Methamphetamine Epidemic Act of 2005 (U.S. Department of Justice 2005), which included provisions for (1) documentation of purchases to allow pharmacies to track sales, (2) storage of the product behind the pharmacy counter or in a locked cabinet, and (3) sales limit of a 30-day supply (this may vary for online or by mail purchases).

### Study limitations

This study has several limitations. Not all loperamide exposures are reported to US PCs; for example, some may be treated in emergency departments or other healthcare settings without a PC being contacted. Therefore, this study underestimates the true number of loperamide exposures in the population. In addition, there may be bias regarding which cases are reported; for example, exposures that are more serious may be more likely to be reported to a PC. Information obtained by PCs is voluntarily self-reported (by the exposed individual, family members, healthcare providers, and others) and cannot be completely verified by the PCs or America’s Poison Centers. Reported cases do not necessarily represent a poisoning or overdose. Although protocols for follow-up and quality control are used, miscoding of data and missing data can occur. Some data are not collected, such as race and ethnicity. The definitions of intentional-misuse and abuse used by the NPDS may differ from those used by other entities and authors, which could limit comparisons among studies (Smith et al. 2013). Despite these limitations, the NPDS is a useful database for comprehensively evaluating the characteristics and trends of loperamide exposures at the national level.

## Conclusions

Although the design of this study precludes determination of causality, there were strong reversals in population-based rate trends for intentional loperamide misuse, abuse, and suspected suicide reported to US PCs that were temporally associated with the loperamide warnings, labeling requirements, and packaging restrictions by the FDA. A temporal association between the FDA actions and reported loperamide cases among young children associated with exploratory behaviors was less apparent. We conclude that the FDA warnings, labeling requirements, and packaging restrictions may have contributed to the observed trend reversal and decrease in reports to US PCs of loperamide cases related to intentional misuse, abuse, and suspected suicide. This demonstrates the potential positive effect that regulatory actions may have on public health. These findings contribute to the evidence supporting the application of similar prevention efforts to reduce poisoning from other medications associated with intentional misuse, abuse, and suicide.

## Data Availability

Data analyzed in this study were from the National Poison Data System, which is a proprietary database owned and managed by America’s Poison Centers. Data requests should be submitted to America’s Poison Centers at: https://www.aapcc.org/national-poison-data-system.

## Abbreviations

CCU: Critical Care Unit
CI: Confidence Interval
FDA: United States Food and Drug Administration
HCF: Health Care Facility
NPDS: National Poison Data System
PC: Poison Center
RR: Risk Ratio
US: United States
